# The relationship between trait mindfulness and subjective wellbeing of kindergarten teachers: The sequential mediating role of emotional intelligence and self-efficacy

**DOI:** 10.3389/fpsyg.2022.973103

**Published:** 2022-09-28

**Authors:** Baocheng Pan, Shiyi Fan, Youli Wang, You Li

**Affiliations:** ^1^College of Education, Wenzhou University, Wenzhou, China; ^2^Suzhou Early Childhood Education College, Suzhou, Jiangsu, China

**Keywords:** trait mindfulness, subjective wellbeing, emotional intelligence, self-efficacy, kindergarten teachers

## Abstract

This study explored the relationship between emotional intelligence and self-efficacy in trait mindfulness and subjective wellbeing. In this study, 323 Chinese kindergarten teachers were measured using the Five Facet Mindfulness Questionnaire, Emotional Intelligence Scale, General Self-efficacy Scale, and Subjective Wellbeing Scale. The study found that subjective wellbeing can be predicted directly from trait mindfulness (β = 0.257, *p* < 0.001). Emotional intelligence could mediate the relationship between trait mindfulness and subjective wellbeing (β = 0.165, *p* = 0.006). Self-efficacy could mediate the relationship between trait mindfulness and subjective wellbeing (β = 0.078, *p* = 0.032). In addition, emotional intelligence and self-efficacy played a sequential mediating role between trait mindfulness and subjective wellbeing (β = 0.072, *p* = 0.005). This study revealed the relationship between kindergarten teachers’ trait mindfulness and subjective wellbeing through structural equation modeling and understood its role path, enriching the research on the Chinese preschool teachers in the field, and providing a literature reference for the international community to understand the Chinese kindergarten teachers. At the same time, the study also has some limitations, such as the use of a cross-sectional design method, a relatively single method, and the impact of COVID-19. However, we believe that this study will further enrich the research literature on the relationship between trait mindfulness and subjective wellbeing of Chinese kindergarten teachers.

## Introduction

In recent years, trends in educational accountability have resulted in more challenging assessments of student performance and greater emphasis on teacher standards. Changes in teacher performance and higher demands on teacher competencies are placing increasing pressure on already overburdened teachers (Spencer et al., 2012; Konstantopoulos, 2014). Consequently, teacher turnover rates are very high in all countries (Hong, 2010). In this context, the philosopher’s claim that happiness is the highest good, the ultimate human drive, is a key point. Therefore, the subject of teachers’ subjective wellbeing has been paid more and more attention by more and more scholars. Subjective wellbeing, as one of the key constructs of positive psychology (Yurayat and Seechaliao, 2021), is often cited as one of the causes of desirable outcomes such as personal physical and mental health (Rohrer and Lucas, 2020). For teachers themselves, high wellbeing and life satisfaction can significantly improve work efficiency and social life relations (Diener, 2009). As one of the reference objects for students’ learning and growth, teachers’ healthy and happy psychological state and explicit behavior will directly affect students’ physical and mental health and learning style (Skaalvik and Skaalvik, 2011). Therefore, the influence and significance of teachers’ subjective wellbeing deserve further study.

According to Bajrami Ollogu and Ismaili (2018), preschool education stage is a very important period of children’s personality development, which produces irreversible mailboxes not only affecting the development of children’s current abilities but also affecting their future success. In preschool education, the quality of education largely depends on a qualified and stable workforce (Sumsion, 2002). Compared with teachers at other education stages, kindergarten teachers, as the main educators who organize and implement educational activities, have a heavy workload and great pressure. In addition to day-to-day behavior management and teaching duties, they also need to deal with non-teaching tasks (McGrath and Huntington, 2007), such as in addition to day-to-day behavior management and teaching duties, they also need to deal with non-teaching tasks (Kelly and Berthelsen, 1995). All kinds of pressures are concentrated in the daily life of kindergarten teachers. If kindergarten teachers lack subjective wellbeing at work, it will lead to a very high turnover rate of kindergarten teachers. Even if there is no resignation, the lack of subjective wellbeing can have a negative impact on the work morale of kindergarten teachers, thereby breaking the attachment relationship between kindergarten teachers and their growth and development (Wells, 2015). In order to promote the sustainable development of preschool education, promote the stability of kindergarten teachers, and improve the subjective wellbeing of kindergarten teachers, this study is particularly urgent.

Existing literature on wellbeing or subjective wellbeing mostly focuses on studying the effects of single factors such as job burnout or work stress on subjective wellbeing (Benevene and Fiorilli, 2015; Wells, 2015). Few literatures have explored the intrinsic relationship with subjective wellbeing from a metacognition perspective. As a positive emotion, subjective wellbeing is bound to have a certain connection with teachers’ own metacognition. At present, although some studies have shown that trait mindfulness has a positive predictive effect on subjective wellbeing (Sahdra et al., 2016), the mechanisms and pathways of trait mindfulness and subjective wellbeing remain unclear. In particular, there is almost no research on the female-dominated group of Chinese preschool teachers, and few studies have focused on the influence mechanism of Chinese kindergarten teachers’ trait mindfulness and subjective wellbeing in the context of the rapid development of preschool education. The current research takes Chinese kindergarten teachers as the object and uses structural equation modeling to reveal the relationship between kindergarten teachers’ trait mindfulness and subjective wellbeing, and to understand its role. We hope that the current research will deepen the research on the subjective wellbeing of preschool teachers in the field of early childhood education. At the same time, this study provides a reference for how to more effectively promote teachers’ subjective wellbeing, so as to provide more information for improving the research on the subjective wellbeing of preschool teachers.

## Literature review and theoretical hypotheses

### Trait mindfulness and subjective wellbeing

As a mode of consciousness, mindfulness is often defined as the full attention paid to what is currently happening in a non-judgmental or accepting manner (Kabat-Zinn, 1990; Brown and Ryan, 2003). At present, mindfulness is commonly used as an intervention as part of the clinical treatment plan to achieve different therapeutic outcomes (Brown et al., 2007). Meanwhile, some studies have shown that in non-clinical samples without experience with meditation or other mindfulness training. This naturally occurring mindfulness has also been significantly correlated with factors such as mental health and behavior regulation (Allen and Kiburz, 2012; Bowlin and Baer, 2012). Individuals with high levels of trait mindfulness can pay attention to the current environment and control their automatic responses, allowing individuals to maintain stable responses in the face of their own particular emotions (Brown et al., 2007).

Subjective wellbeing is a general term that describes the level of happiness people experience based on their emotional and cognitive assessments of life (Diener and Ryan, 2009). As a measure of social and personal quality of life (Diener et al., 2003), subjective wellbeing has multiple dimensions, including life satisfaction, job satisfaction, positive influence, and happy mood (Diener, 2000). There is growing evidence that high levels of subjective wellbeing and life satisfaction can significantly improve an individual’s daily life, including physical wellbeing, social harmony, and economic performance (Lyubomirsky et al., 2005).

The Affective Cognition Theory (Blore et al., 2011) believes that subjective wellbeing is mainly affected by trait emotion, and trait emotion is influenced by acceptance cognition and personality. Naturally occurring mindfulness, as a trait attribute, can also be indirectly influenced by personality and cognition by regulating one’s own behavior and psychology (Allen and Kiburz, 2012; Bowlin and Baer, 2012). This may have a more positive effect on subjective wellbeing. For example, higher levels of mindfulness are associated with better outcomes such as mental health, relationship satisfaction, and pain management (Brown et al., 2007). Research by Shier and Graham (2011) argues that mindfulness affects social workers’ subjective wellbeing, that is, high levels of mindfulness in practice settings can help people perceive wellbeing. In the context of the COVID-19 pandemic era, research has shown that Mindfulness-Based Stress Reduction program (MBSR) can be effective in reducing the negative psychology associated with isolation and social distancing, as well as improving teacher resilience and wellbeing (Matiz et al., 2020).

Therefore, this study proposes the hypothesis:

H1: Trait mindfulness can positively predict subjective wellbeing.

### Trait Mindfulness, emotional intelligence, and subjective wellbeing

Emotional intelligence is the ability to monitor one’s own and others’ emotions and differentiate between different emotions to guide one’s thinking and actions (Mayer and Salovey, 1997). Existing research shows that emotional intelligence can predict an individual psychological and behavioral abilities to a certain extent, so this research finding has been widely in education and psychology (Mayer et al., 2008). In the field of education, especially preschool, as a key stage in the development of children’s core competencies, teachers with high emotional intelligence are required to demonstrate their positive behaviors (Ciucci et al., 2015), thereby developing their emotional competencies and social styles (Fernández-Molina et al., 2019).

According to the sociogenomic model of personality (Roberts and Jackson, 2008), personality traits can not only directly affect an individual’s behavior outcomes, but indirectly explain specific behaviors through cognition. Specifically, trait mindfulness, as a personality trait, can indirectly affect subjective wellbeing by affecting emotional intelligence. Additionally, other research suggests that emotional intelligence is associated with trait mindfulness. For example, a number of studies such as Sinclair and Feigenbaum (2012) have speculated that the higher the level of mindfulness in an individual, the higher the emotional intelligence. In the research on the influencing factors of college students’ subjective wellbeing, it is found that trait mindfulness can indeed affect subjective wellbeing through mediating factors such as core self-evaluation and emotional intelligence (Schutte and Malouff, 2011). Mindfulness can encourage individuals to accurately perceive emotions and effectively regulate their own and others’ emotions, thereby promoting the development of emotional competence (Chambers et al., 2009).

In job demand-resource theory, emotional intelligence is regarded as a personal resource that functions similarly to job resources (Schaufeli and Taris, 2014). At the same time, personal resources are also regarded as one of the predictors of happiness (Mérida-López et al., 2020). At present, a large number of studies have shown that there is a certain correlation between emotional intelligence and emotional wellbeing (Koydemir et al., 2013; Liu et al., 2013). For example, Ferragut and Fierro (2012) found a significant correlation between emotional intelligence, academic achievement, and subjective wellbeing. Rey et al. (2016) found that teachers’ emotional intelligence is closely related to burnout and teacher job satisfaction. To a certain extent, special education teachers with higher emotional intelligence are better at using rational evaluation and emotion regulation, thereby enhancing subjective wellbeing (Li and Zhang, 2021). Trait mindfulness can better promote the development of emotional intelligence, which in turn affects the subjective wellbeing of individuals.

Therefore, the following hypotheses are proposed in this study:

H2: Emotional intelligence plays a mediating role in trait mindfulness and subjective wellbeing.

H2a: Trait mindfulness is positively correlated with emotional intelligence.

H2b: There is a significant positive correlation between emotional intelligence and subjective wellbeing.

### Trait Mindfulness, self-efficacy, and subjective wellbeing

Self-efficacy is a self-judgment of a person’s ability to perform tasks in a particular domain (Bandura, 2010), and is a sense of efficiency, competence, and ability to cope with life (Bandura and Schunk, 1981). Studies on the antecedents of self-efficacy has shown that cognitive ability (Phillips and Gully, 1997), experience (Judge et al., 2007) and personal characteristics can predict levels of self-efficacy (Thoms et al., 1996). Teachers’ self-efficacy can be understood as the belief that teachers can influence students’ self-confidence to a certain extent (Muijs and Reynolds, 2015). Some scholars have found that kindergarten teachers generally have sufficient self-efficacy beliefs, and this ability will affect the classrooms management, teaching process and communication skills of kindergarten teachers (Kırkıç and Çetinkaya, 2020).

According to self-efficacy theory (Bandura, 1994), self-efficacy is a motivational concept that involves a person’s perception of one’s personal abilities (Tschannen-Moran and Hoy, 2007). For example, in a study by Judge et al. (2007) showed that personal factors such as five major characteristics and psychological abilities have a positive effect on self-efficacy. In fact, from a metacognitive perspective, as a personal trait, mindfulness has a positive effect on self-efficacy. In addition, a study on physical education and mindfulness of college students found that college students can develop mindfulness through physical education courses, which can have a certain impact on self-efficacy (Caldwell et al., 2010). Luberto et al. (2014) suggests that stronger coping self-efficacy is associated with mindfulness, leading to the regulation of negative emotions. Hanley et al. (2015a) research showed that mindfulness and positive reassessment were positively correlated with academic self-efficacy after perceived failure.

According to social cognitive theory, a strong sense of self-efficacy is related to the way people think and behave, and in many ways enhances an individual’s sense of achievement and wellbeing (Bandura, 1994). Self-efficacy determines the form and intensity of emotional responses to events deemed critical to people, thereby influencing their perceptions. In past studies, self-efficacy has been associated with wellbeing and power processes as a form and basic need for human wellbeing (Loton and Waters, 2017). Self-efficacy is an important predictor of subjective wellbeing (Ngui and Lay, 2019). In education, many studies have shown that teacher self-efficacy is related to many beneficial outcomes, including teacher motivation and wellbeing and its various dimensions (Flores, 2006). For example, teachers with high levels of self-efficacy have higher job satisfaction (Barni et al., 2019), which is closely related to subjective wellbeing. Dicke et al. (2015) and others found that for individual teachers, personal characteristics similar to self-efficacy can alleviate their stress and further enhance teachers’ subjective wellbeing. In addition to personal attributes, self-efficacy acquired in teaching activities can also negatively predict teacher burnout (Lauermann and König, 2016).

Therefore, this study proposed the following hypothesis:

H3: Self-efficacy plays a mediating role in trait mindfulness and subjective wellbeing.

H3a: Trait mindfulness is significantly positively correlated with self-efficacy.

H3b: There is a significant positive correlation between self-efficacy and subjective wellbeing.

### Emotional intelligence and self-efficacy

More and more studies have found that teachers’ behavior is not only influenced by cognitive factors, but also by non-cognitive factors (Korthagen and Evelein, 2016). Self-efficacy, as a predictor of cognitive participation (Walker et al., 2006), has certain effects on teachers at all stages. For example, sufficient self-efficacy beliefs can have a certain planning and adjustment effect on the teaching process, communication skills and classroom management of kindergarten teachers (Kırkıç and Çetinkaya, 2020). Emotional intelligence, as a non-cognitive affective factor, also plays an important role. Studies have found that teachers with high emotional intelligence adopt more rational strategies to solve and manage classroom conflicts (Valente and Lourenço, 2020). Meanwhile, other studies have revealed the correlation between emotional intelligence and self-efficacy. For example, Judge et al. (2007) found that emotional intelligence can further contribute to the formation of self-efficacy through emotional self-regulation and self-awareness. Kostiæ-Bobanoviæ (2020) revealed a significant positive correlation between foreign language teachers’ emotional performance and self-efficacy. Chan (2004) also found a strong correlation between emotional intelligence and self-efficacy beliefs for most teachers.

According to cognitive-affective theory (Mischel and Shoda, 1995), events encountered by individuals interact with the complex cognitive-affective units in the personality system to influence behavioral outcomes. Among them, the emotions felt by individuals have a significant impact on social information processing and behavior processing, because the processing of important information (such as self-cognitive beliefs) often has the function of emotional arousal. For example, Bandura (1997) believes that self-awareness and emotion regulation are crucial to the development of self-efficacy, and individuals with higher self-awareness and emotion control ability may have higher self-efficacy. Cohen and Abedallah (2015) found that emotional intelligence can influence self-efficacy by influencing causal reasoning or emotion for coping with important outcomes at work. In particular, Wu et al. (2019) found that emotional intelligence can improve teachers’ self-efficacy through the appropriate use of emotional skills. In addition, studies in interpersonal communication have found that teachers with emotional intelligence can build satisfying relationships with others, facilitate a person’s achievement of goals, and influence self-efficacy (Hassan et al., 2013).

Therefore, this study proposed the following hypothesis:

H4: Emotional intelligence is significantly positively correlated with self-efficacy.

H5: Emotional intelligence and self-efficacy play serial mediating roles in trait mindfulness and subjective wellbeing.

**FIGURE 1:**
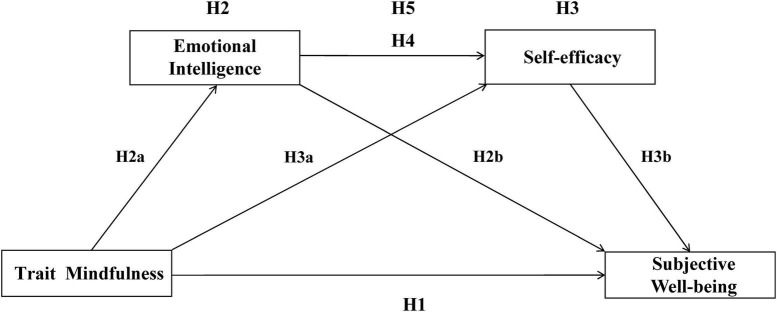
Theoretical hypothesis.

Theoretical hypothesis see Figure 1.

## Materials and methods

### Participants

In this study, a cluster random sampling method was used to conduct a sample survey of 13 kindergartens in 4 districts of Jinan, Shandong Province, China. The survey was conducted from April to May 2022. The formal implementation is in charge of preschool education interns who have received unified training. After communicating with the kindergarten person in charge and obtaining their approval, the questionnaire was distributed on the spot through a paper-and-pencil test. Each test adopts uniform guidelines and emphasizes the confidentiality of the survey to ensure the validity and authenticity of the questionnaire. A total of 350 questionnaires were distributed, of which 346 were actually recovered. For questionnaires with accurate data, we use the direct elimination method. Finally, 323 valid questionnaires were recovered, and the effective recovery rate was 92.29%.

The kindergarten teachers participating in this survey are all full-time in-service teachers. Among them, there are 7 were male teachers, accounting for 2.20%, and 316 female teachers, accounting for 97.80%. Teachers aged 25–27 accounted for 84.80%, teachers aged 28–30 accounted for 6.20%, teachers aged 31–33 accounted for 5.60%, and teachers aged 34–36 accounted for 3.40%. Teachers with a college degree accounted for 72.80%, and the proportion of teachers with undergraduate degrees is 27.20%. See Table 1.

**TABLE 1:** Social demographic features of participants (*N* = 323).

Variables		Percentages
Gender	Male	2.20%
	Female	97.80%
Age	25–27	84.80%
	28–30	6.20%
	31–33	5.60%
	34–36	3.40%
Educational background	Junior college	72.80%
	Undergraduate course	27.20%

This study was ethically reviewed and approved by the Research Ethics Committee of Wenzhou University. Before analysis, the kurtosis and skewness of the data were tested, and the main scales distribution shows enough normality indices, which allows us to take this into consideration for more in-depth statistical inferential analyses.

### Measure

#### Five facet mindfulness questionnaire

Five Facet Mindfulness Questionnaire (Baer et al., 2006) is a 39-item questionnaire that measures trait mindfulness. This questionnaire has five dimensions, including observing, describing, acting with awareness, non-judgment, and non-reacting (Pang and Ruch, 2019). A sample item was, “When I do things, my mind wanders off and I’m easily distracted.” The Chinese version of the Five Facet Mindfulness Questionnaire was translated by Deng et al. (2011) and has been shown to have good reliability and validity in the Chinese population. A five-point Likert-type scale was used to assess the frequency that each item was correct to respondents (1 = never or very rarely true and 5 = very often or always true). In this study, the Cronbach’s α value of the scale was 0.953, and the Cronbach’s α values of the sub-dimension scales were 0.894, 0.878, 0.920, 0.851, and 0.892, respectively.

#### Emotional intelligence scale

Emotional Intelligence Scale developed by Wong and Law (2002) is widely used to measure emotional intelligence. This scale measures four dimensions of emotional intelligence, including self-emotion appraisal (SEA), others’ emotion appraisal (OEA), use of emotion (UOE), and regulation of emotion (ROE). The Chinese version translated by Law et al. (2004) as good reliability in Chinese modification and proof. There are 16 items in the scale (e.g., “I can control my temper when I meet difficulties”), and seven points are used (1 = strongly disagree and 7 = strongly agree). In this study, the Cronbach’s α value of the scale was 0.912, and the Cronbach’s α values of the sub-dimensional scale were 0.889, 0.851, 0.902, and 0.857, respectively.

#### General self-efficacy scale

The General Self-efficacy Scale was developed by Scholz et al. (2002), which has been widely used in the world. In China, this scale has been proven to have good reliability and validity (Zhang and Schwarzer, 1995). The General Self-efficacy Scale consists of 10 items (e.g., “I can handle whatever comes my way”), using four point score (1 = completely is not correct and 4 = all correct). In this study, the Cronbach’s α value of the scale was 0.877.

#### Subjective wellbeing scale

The Subjective Wellbeing Scale used in this study was the life satisfaction scale compiled by Diener (1984) to measure the cognitive dimension of subjective wellbeing, with a total of five items. The scale has good applicability in Chinese subjects (Ma and Sun, 2009). The scale adopts Likert’s 7-point score (1 = very disagree and 7 = very agree). A sample item was, “I am satisfied with the conditions of my life.” In this study, the Cronbach’s α value of the scale was 0.811.

### Statistical analyses

This study uses SPSS 22.0 and Mplus version 8.3 for data analysis, in which SPSS is mainly used for data sorting, descriptive statistical analysis, etc. Mplus is mainly used for model inspection. Participants who lack descriptive data or many data points are processed by list deletion when running the analysis. In the analysis, teachers’ gender, age, and educational background are taken as control variables.

## Results

### Test of common method deviation

Using Harman single factor test method, 13 factors with characteristic root greater than 1 were obtained. The explanation rate of the first factor is 24.92%, which is less than the cut-off value of 40% (Podsakoff et al., 2003), indicating that there is no significant common method bias in this study.

### Descriptive statistical analysis

Table 2 lists the major variables and Pearson correlation coefficients between each dimension. As can be seen from Table 2, subjective wellbeing is significantly positively correlated with all dimensions of trait mindfulness, all dimensions of emotional intelligence, and self-efficacy. According to the views of Tsui et al. (1995), the critical value of the correlation level with serious multicollinearity problems is generally more than 0.75. In this study, the correlation coefficient of all variables is less than 0.70, and there is no serious multicollinearity problem among the major variables.

**TABLE 2:** Means, standard deviations, and correlations of the major study variables.

Variable	M	SD	1	2	3	4	5	6	7	8	9	10	11	12	13	14
1. Gender	0.98	0.15	1													
2. Age	27.05	2.35	−0.015	1												
3. Educational background	1.27	0.45	0.091	−0.06	1											
4. Observe	3.18	0.80	0.09	0.046	−0.032	1										
5. Describe	3.43	0.73	0.058	0.056	−0.012	0.532**	1									
6. ActAware	3.42	0.92	0.077	0.051	−0.001	0.532**	0.586**	1								
7. Non-judge	3.24	0.67	0.096	0.049	0.104	0.590**	0.510**	0.430**	1							
8. Non-react	3.35	0.84	0.131*	0.067	−0.004	0.509**	0.509**	0.668**	0.498**	1						
9. SEA	4.39	1.28	−0.03	0.042	0.022	0.110*	0.278**	0.196**	0.248**	0.214**	1					
10. OEA	4.26	1.38	−0.003	0.053	0.140*	0.262**	0.324**	0.238**	0.298**	0.284**	0.497**	1				
11. UOE	4.56	1.34	−0.009	−0.01	0.056	0.278**	0.214**	0.297**	0.122*	0.133*	0.538**	0.420**	1			
12. ROE	4.53	1.27	−0.056	−0.032	0.047	0.248**	0.231**	0.190**	0.300**	0.219**	0.480**	0.454**	0.359**	1		
13. SE	2.59	0.51	0.03	0.084	−0.003	0.263**	0.234**	0.206**	0.248**	0.158**	0.314**	0.208**	0.267**	0.209**	1	
14. SWB	3.90	0.94	0.034	0.118*	0.017	0.300**	0.413**	0.313**	0.313**	0.280**	0.364**	0.316**	0.288**	0.251**	0.439**	1

**p < 0.01, *p < 0.05. SEA, Self-emotion appraisal; OEA, Others’ emotion appraisal; UOE, Use of emotion; ROE, Regulation of emotion; SE, Self-efficacy; SWB, Subjective wellbeing. Gender is the dummy variable (0 = male, 1 = female).

### Model inspection

The model was fitted with Mplus, and the fitting index of the model was ML χ^2^ = 462.058, *df* = 309, χ^2^/*df* = 1.495, CFI = 0.946, TFI = 0.940, RMSEA = 0.039, SRMR = 0.043. All the indicators are within the acceptable range, and the model is ideal (see Table 3).

**TABLE 3:** Fit indices of the model.

Fit indices	Recommended threshold	Scores	Remarks
ML χ^2^	–	462.058	–
Df	–	309	–
χ^2^/df	1 < χ^2^/df < 3	1.495	Acceptable
CFI	>0.9	0.946	Acceptable
TLI	>0.9	0.940	Acceptable
RMSEA	<0.08	0.039	Acceptable
SRMR	<0.08	0.043	Acceptable

### The significance test of mediating effect

On the basis of good model fitting, the Bootstrap program of Mplus was used to repeat the sample 5,000 times. The results show that the path coefficients of trait mindfulness, emotional intelligence, self-efficacy, and subjective wellbeing are all significant.

Trait mindfulness is positively related to subjective wellbeing (β = 0.257, *p* < 0.001), supporting H1. Trait mindfulness is positively related to emotional intelligence (β = 0.461, *p* < 0.001), supporting H2a. Emotional intelligence is positively related to subjective wellbeing (β = 0.250, *p* = 0.001), supporting H2b. Trait mindfulness is positively related to self-efficacy (β = 0.163, *p* = 0.02), supporting H3a. Self-efficacy is positively related to subjective wellbeing (β = 0.333, *p* < 0.001), supporting H3b. Emotional intelligence is positively related to self-efficacy (β = 0.328, *p* < 0.001), supporting H4 (see Table 4).

**TABLE 4:** The direct effect of the research paths and research model hypothesis analysis.

DV	IV	Std. est.	S.E.	Est./S.E.	*P*-value	R^2^	Hypo and path	Remarks
SWB	TM	0.257	0.069	3.752	***	0.429	H1: TM → SWB	Support
	EI	0.250	0.074	3.361	0.001		H2b: EI → SWB	Support
	SE	0.333	0.070	4.766	***		H3b: SE → SWB	Support
EI	TM	0.461	0.065	7.038	***	0.228	H2a: TM → EI	Support
SE	TM	0.163	0.070	2.326	0.020	0.187	H3a: TM → SE	Support
	EI	0.328	0.072	4.568	***		H4: EI → SE	Support

***p < 0.001. TM, Trait mindfulness; EI, Emotional intelligence.

Table 5 shows the indirect effects of the study path. Emotional intelligence mediates the relationship between trait mindfulness and subjective wellbeing (β = 0.165, *p* = 0.006), with 95% confidence interval [0.069–0.311], excluding 0, supporting H2. The mediating effect of emotional intelligence between trait mindfulness and subjective wellbeing accounted for 24.09%.

**TABLE 5:** The indirect effect of the research paths.

Path	Std. est.	S.E.	Est./S.E.	*P*-value	Boot LLCI	Boot ULCI	The proportion of the effect
H2: TM → EI → SWB	0.165	0.060	2.767	0.006	0.069	0.311	24.09%
H3: TM → SE → SWB	0.078	0.036	2.145	0.032	0.015	0.163	11.39%
H5: TM → EI → SE → SWB	0.072	0.026	2.813	0.005	0.035	0.142	10.51%
TOTALIND	0.316	0.071	4.467	***	0.194	0.474	46.13%
TOTAL	0.685	0.104	6.581	***	0.49	0.895	100.00%

***p < 0.001.

Self-efficacy mediates the relationship between trait mindfulness and subjective wellbeing (β = 0.078, *p* = 0.032), with 95% confidence interval [0.015–0.163], excluding 0, supporting H3. The mediating effect of self-efficacy between trait mindfulness and subjective wellbeing accounted for 11.39%.

**FIGURE 2:**
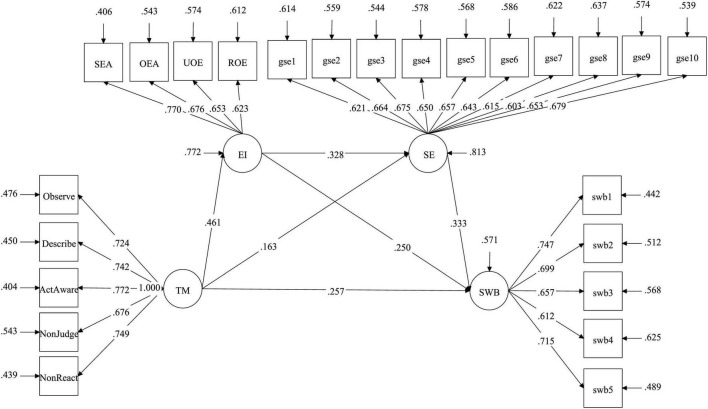
Structural equation model.

Emotional intelligence and self-efficacy sequentially mediate the relationship between trait mindfulness and subjective wellbeing (β = 0.072, *p* = 0.005), with 95% confidence interval [0.035–0.142], excluding 0, supporting H5. The sequential mediation of emotional intelligence and self-efficacy between trait mindfulness and subjective wellbeing accounted for 10.51% (see Figure 2).

## Discussion

### Trait mindfulness can positively predict subjective wellbeing

This study found that trait mindfulness significantly positively predicted the subjective wellbeing of kindergarten teachers, which is consistent with the research hypothesis and consistent with previous studies (Hanley et al., 2015b). The findings confirm that subjective wellbeing is one of the most relevant factors of trait mindfulness (Brown et al., 2009). At the same time, the research also further verified the affective cognition theory (Davern et al., 2007), which holds that subjective wellbeing is related to trait attributes, and can also be related to receptive cognition and personality through trait attributes. Mindfulness that arises naturally has trait attributes, is related to personality and cognition, and has the function of regulating an individual’s mental state and explicit behavior, thereby enhancing subjective wellbeing. The higher the level of individual trait mindfulness of kindergarten teachers, the higher the level of self-cognition and self-acceptance in the teaching process, resulting in higher subjective wellbeing. On the contrary, the lower the level of trait mindfulness, the more critical cognition, which is not conducive to the production of subjective wellbeing.

### Emotional intelligence plays a mediating role in trait mindfulness and subjective wellbeing

This study further explored the mediating role of emotional intelligence and self-efficacy between trait mindfulness and subjective wellbeing. Firstly, this study found that the mediating role of emotional intelligence was particularly prominent, accounting for 24.09%, much higher than self-efficacy (11.39%). This result suggests that trait mindfulness is more likely to be associated with the mediation of emotional intelligence in subjective wellbeing. Because a high level of mindfulness can experience the immediate clarity and vividness of the current environment (Brown et al., 2007). People who are highly mindful can play a role in staying focused in the present moment. Similarly, in terms of emotion regulation, the level of self-regulation in mindfulness has a certain correlation with the emotion management dimension of emotional intelligence (Schutte and Malouff, 2011). Specifically, individuals with a high level of mindfulness can not only clearly perceive their own emotional fluctuations in the process of social interaction but also closely observe the emotions of others, which is conducive to timely stop the behaviors caused by bad emotions. When an person is able to control and use their emotions well, there are some positive outcomes.

On the contrary, with low levels of trait mindfulness, individuals cannot control their emotions well and achieve correspondingly positive outcomes. This also confirms the Job Demands-Resources model (JD-R model; Schaufeli and Taris, 2014), which holds that emotional intelligence, as a kind of personal resource, is regarded as one of the factors to predict happiness. In the long-term teaching process of kindergarten teachers, their emotional perception, understanding and management will have a certain impact on the growth and development of children. However, kindergarten teachers with high levels of mindfulness can process their emotions effectively. Such high personal resources will lead to the improvement of motivation and productivity (Schaufeli and Taris, 2014), prompting kindergarten teachers to continually generate better parenting approaches and a continued passion for education. Ultimately, the individuals will achieve a high degree of subjective wellbeing. Thus, trait mindfulness can be related to the subjective wellbeing of kindergarten teachers through emotional intelligence.

### Self-efficacy plays a mediating role in trait mindfulness and subjective wellbeing

Meanwhile, self-efficacy also played a significant mediating role in the relationship between trait mindfulness and subjective wellbeing, with the mediating effect accounting for 11.39%. This is because the quality of mindfulness reduces personal distrust and judgmental thoughts and prevent the current situation from being influenced by the past and the future (Bayır and Aylaz, 2021). People with high levels of mindfulness are more likely to achieve this goal, thus achieving higher self-efficacy. Higher self-efficacy leads to a change in mindset and increased mobility, which can help solve life’s difficulties. Happiness comes when these positive experiences build up in your life. According to social cognitive theory (Bandura, 1986), self-efficacy can determine the form and intensity of people’s emotional responses to events that are critical to people, thereby affecting their cognition and expected behavior. This also applies to the teaching process of kindergarten teachers. Kindergarten teachers’ high levels of trait mindfulness can reduce their judgmental thinking, focus on course content during teaching, and gain high levels of self-efficacy, which in turn leads to higher subjective wellbeing. Therefore, trait mindfulness can be correlated with the subjective wellbeing of kindergarten teachers by increasing levels of self-efficacy. The accumulation of good self-efficacy in the teaching process will bring about the recovery of teachers’ teaching enthusiasm and solve the problem of high turnover rate of kindergarten teachers.

### Emotional intelligence and self-efficacy play serial mediating roles in trait mindfulness and subjective wellbeing

Finally, the most important part of this study is to reveal the sequential mediating effects of emotional intelligence and self-efficacy on trait mindfulness and subjective wellbeing, accounting for 10.51%. This ratio is very close to the effect value of the single variable of the self-efficacy univariate, revealing that emotional intelligence can not only directly related to subjective wellbeing but also has a high degree of closeness to self-efficacy. This is because emotional intelligence mainly emphasizes the individual’s perception and regulation of their own emotions and emotional states, and plays a key role in the formation of self-efficacy (Bandura et al., 1999). This result further validates the cognitive-emotion theory (Mischel and Shoda, 1995). We can find that kindergarten teachers with high emotional intelligence have the ability to perceive and manage emotions, and can meet the corresponding needs of children in all aspects of daily life and curriculum content, thus demonstrating complete self-confidence, which will inevitably increase the sense of individual self-efficacy. On the other hand, if the emotional intelligence level of teachers is not high, and does not pay attention to the cultivation of emotional intelligence level, the result will not only reduce self-efficacy, but also produce a series of negative effects.

## Conclusion

The results of this study suggest that trait mindfulness can directly predict subjective wellbeing. Trait mindfulness can not only predict the level of subjective wellbeing through emotional intelligence but also correlates with subjective wellbeing through self-efficacy. We also found that emotional intelligence and subjective wellbeing play a serial mediating role in trait mindfulness and subjective wellbeing.

## Research contributions, limitations, and future research directions

This study has made some theoretical contributions. The existing literature has rarely paid attention to the mechanism and path of action of trait mindfulness and subjective wellbeing, especially the related research on Chinese kindergarten teachers is almost blank. The current research reveals the relationship between kindergarten teachers’ trait mindfulness and subjective wellbeing through the structural equation model and understands its role path, which provides research literature and research reference for the research on kindergarten teachers’ subjective wellbeing, and also provides a basis for the research on kindergarten teachers’ wellbeing. In addition, the greatest contribution of the article is to provide a literature reference for the international community to understand the trait mindfulness and subjective wellbeing of Chinese kindergarten teachers.

This study also brings us relevant practical enlightenment. In recent years, several studies have found that mindfulness-based stress reduction training can effectively improve the level of individual mindfulness. Therefore, in addition to daily educational skills training, kindergartens should also provide kindergarten teachers with courses on mindfulness training or mindfulness intervention. However, due to the update of novel coronavirus, the world has entered a post-COVID-19 era. If professional offline mindfulness courses cannot be provided due to some objective factors, online mindfulness interventions have also been shown to improve mindfulness levels and mental health effects. In addition, this study found that emotional intelligence, as a mediator, not only affects subjective wellbeing but is also closely related to self-efficacy. Therefore, we must pay attention to and take effective measures to pay attention to the emotional intelligence level of kindergarten teachers. For example, strengthen humanistic care in kindergartens and adjust the cognitive model. Among kindergarten teachers, the self-efficacy of kindergarten teachers should be enhanced. For example, as a preschool education leader, teachers should be given more encouragement and support, so that kindergarten teachers can feel their competence in educational work. In addition, the professional knowledge and training of teachers should be strengthened, and the ability to organize activities should be enhanced, so as to better generate self-efficacy.

Future research can be improved and expanded from the following four aspects. First, a cross-sectional design method was adopted in this study. Although this study is based on sufficient theory and empirical evidence, coupled with high-reliability measurement scales, the cross-sectional study inevitably has certain limitations. For example, cross-sectional models do not allow causal inference. Cross-sectional analysis cannot model stable relationships between variables over time. In other words, the cross-sectional analysis is unable to determine the extent to which correlations between measures reflect an influence of one measure on another over time or instead reflect ongoing stable relations between measures. Relative to longitudinal designs, cross-sectional analyses have higher bias in parameter estimates (Maxwell and Cole, 2007). In the future, the longitudinal design method can be used to explore the relationship between trait mindfulness and subjective wellbeing. Follow-up research can be conducted on kindergarten teachers in China, focusing on the changes and influence of kindergarten teachers’ trait mindfulness and subjective wellbeing. Second, the scale data in this study were derived from self-report, which is subjective. In the future, mixed research methods, such as experimental methods or qualitative research, can be used. The subjective wellbeing of kindergarten teachers will be affected by events. Although the quantitative research method used in the current study can measure the current subjective wellbeing of kindergarten teachers, it is impossible to know the specific context of the relationship between trait mindfulness and kindergarten teachers’ subjective wellbeing. In particular, the findings suggest that emotional intelligence is much more mediating than self-efficacy in this relationship. The specific reasons for this phenomenon cannot be verified by quantitative research. In the future, we can consider combining interviews or field investigations to understand the relationship between kindergarten teachers’ traits mindfulness, emotional intelligence, self-efficacy, and subjective wellbeing. Third, the main group of this study is Chinese kindergarten teachers, but women account for the vast majority of Chinese kindergarten teachers, and our research sample also reflects this phenomenon. Although the current data showed that gender was not significantly associated with the variables in the study, gender was also included as a control variable in the study. However, it is necessary to conduct a comparative study of the variables in this study between Chinese kindergarten teachers and women in the future, and even an international comparative study, and we believe that this study may have exciting findings. In addition, the data collected in this study are susceptible to the force majeure of COVID-19, which may have some impact on the wellbeing of individuals in charge. Comparative studies could be considered in the future after the COVID-19 is over or well-controlled. Finally, this study only considered emotional intelligence and self-efficacy as mediating factors. However, there are many factors that affect kindergarten teachers’ subjective wellbeing, and the mechanism of other mediating variables between trait mindfulness and subjective wellbeing can be considered in the future.

## Data availability statement

The datasets generated for this study are available on request to the corresponding author.

## Ethics statement

The studies involving human participants were reviewed and approved by the Research Ethics Committee of Wenzhou University. The patients/participants provided their written informed consent to participate in this study.

## Author contributions

BP designed, prepared, carried out the data collection process, and wrote the article. SF analyzed and verified the data in this article. YW searched the literature and revised the manuscript. YL revised the section of the analysis and discussion and corrected the entire manuscript. All authors contributed to the article and approved the submitted version.

## References

[B1] Allen T. D. Kiburz K. M. (2012). Trait mindfulness and work-family balance among working parents: The mediating effects of vitality and sleep quality. *J. Vocat. Behav.* 80 372–379. 10.1016/j.jvb.2011.09.002

[B2] Baer R. A. Smith G. T. Hopkins J. Krietemeyer J. Toney L. (2006). Using self-report assessment methods to explore facets of mindfulness. *Assessment* 13 27–45. 10.1177/1073191105283504 16443717

[B3] Bajrami Ollogu E. Ismaili D. (2018). The impact of pre-school education in the next levels of educational system case study the city of skopje. *Knowl. Int. J.* 28 871–875. 10.35120/kij2803871e 27599931

[B4] Bandura A. (1986). *Social foundations of thought and action: A social cognitive theory / Albert Bandura.* Hoboken, NJ: Prentice-Hall.

[B5] Bandura A. (1994). “Bandura self-efficacy defined,” in *Encyclopedia of human behavior.* New York, NY: Academic Press

[B6] Bandura A. (1997). *Self-efficacy: The exercise of control.* New York, NY: W.H. Freeman and Company. American Psychological Association, 23.

[B7] Bandura A. (2010). *Self-efficacy–Bandura. The corsini encyclopedia of psychology.* Hoboken, NJ: Wiley.

[B8] Bandura A. Schunk D. H. (1981). Cultivating competence, self-efficacy, and intrinsic interest through proximal self-motivation. *J. Pers. Soc. Psychol.* 41 586–598. 10.1037/0022-3514.41.3.586

[B9] Bandura A. Freeman W. H. Lightsey R. (1999). Self-efficacy: The exercise of control. *J. Cogn. Psychother.* 13 158–166. 10.1891/0889-8391.13.2.158 11261958

[B10] Barni D. Danioni F. Benevene P. (2019). Teachers’ self-efficacy: The role of personal values and motivations for teaching. *Front. Psychol.* 10:1645. 10.3389/fpsyg.2019.01645 31354604 PMC6640028

[B11] Bayır B. Aylaz R. (2021). The effect of mindfulness-based education given to individuals with substance-use disorder according to self-efficacy theory on self-efficacy perception. *Appl. Nurs. Res.* 5:151354. 10.1016/j.apnr.2020.151354 32907766

[B12] Benevene P. Fiorilli C. (2015). Burnout syndrome at school: A comparison study with lay and consecrated italian teachers. *Mediterr. J. Soc. Sci.* 6 501–506. 10.5901/mjss.2015.v6n1p501

[B13] Blore J. D. Stokes M. A. Mellor D. Firth L. Cummins R. A. (2011). Comparing multiple discrepancies theory to affective models of subjective wellbeing. *Soc. Indic. Res.* 100 1–16. 10.1007/s11205-010-9599-2

[B14] Bowlin S. L. Baer R. A. (2012). Relationships between mindfulness, self-control, and psychological functioning. *Pers. Individ. Differ.* 52 411–415. 10.1016/j.paid.2011.10.050

[B15] Brown K. W. Ryan R. M. (2003). The benefits of being present: Mindfulness and its role in psychological well-being. *J. Pers. Soc. Psychol.* 84 822–848. 10.1037/0022-3514.84.4.822 12703651

[B16] Brown K. W. Kasser T. Ryan R. M. Alex Linley P. Orzech K. (2009). When what one has is enough: Mindfulness, financial desire discrepancy, and subjective well-being. *J. Res. Pers.* 43 727–736. 10.1016/j.jrp.2009.07.002

[B17] Brown K. W. Ryan R. M. Creswell J. D. (2007). Mindfulness: Theoretical foundations and evidence for its salutary effects. *Psychol. Inq.* 18 211–237. 10.1080/10478400701598298

[B18] Caldwell K. Harrison M. Adams M. Quin R. H. Greeson J. (2010). Developing mindfulness in college students through movement-based courses: Effects on self-regulatory self-efficacy, mood, stress, and sleep quality. *J. Am. Coll. Health* 58 433–442. 10.1080/07448480903540481 20304755 PMC2879280

[B19] Chambers R. Gullone E. Allen N. B. (2009). Mindful emotion regulation: An integrative review. *Clin. Psychol. Rev.* 29 560–572. 10.1016/j.cpr.2009.06.005 19632752

[B20] Chan D. W. (2004). Perceived emotional intelligence and self-efficacy among Chinese secondary school teachers in Hong Kong. *Pers. Individ. Differ.* 36 1781–1795. 10.1016/j.paid.2003.07.007

[B21] Ciucci E. Baroncelli A. Toselli M. (2015). Meta-emotion philosophy in early childhood teachers: Psychometric properties of the Crèche educator emotional styles questionnaire. *Early Child. Res. Q.* 33 1–11. 10.1016/j.ecresq.2015.04.006

[B22] Cohen A. Abedallah M. (2015). The mediating role of burnout on the relationship of emotional intelligence and self-efficacy with ocb and performance. *Manage. Res. Rev.* 38 2–28. 10.1108/MRR-10-2013-0238

[B23] Davern M. T. Cummins R. A. Stokes M. A. (2007). Subjective wellbeing as an affective-cognitive construct. *J. Happiness Stud.* 8 429–449. 10.1007/s10902-007-9066-1

[B24] Deng Y. Q. Liu X. H. Rodriguez M. A. Xia C. Y. (2011). The five facet mindfulness questionnaire: Psychometric properties of the Chinese version. *Mindfulness* 2 123–128. 10.1007/s12671-011-0050-9

[B25] Dicke T. Parker P. D. Holzberger D. Kunina-Habenicht O. Kunter M. Leutner D. (2015). Beginning teachers’ efficacy and emotional exhaustion: Latent changes, reciprocity, and the influence of professional knowledge. *Contemp. Educ. Psychol.* 41 62–72. 10.1016/j.cedpsych.2014.11.003

[B26] Diener E. (1984). Subjective well-being. *Psychol. Bull.* 95 542–575. 10.1037/0033-2909.95.3.542 6399758

[B27] Diener E. (2000). Subjective well-being: The science of happiness and a proposal for a national index. *Am. Psychol.* 55 34–43. 10.1037/0003-066X.55.1.34 11392863

[B28] Diener E. (2009). *Assessing well-being. The collected works of ed diener.* New York, NY: Springer, 10.1007/978-90-481-2354-4

[B29] Diener E. Ryan K. (2009). Subjective well-being: A general overview. *S. Afr. J. Psychol.* 39 391–406. 10.1177/008124630903900402

[B30] Diener E. Oishi S. Lucas R. E. (2003). Personality, culture, and subjective well-being: Emotional and cognitive evaluations of life. *Ann. Rev. Psychol.* 54 403–425. 10.1146/annurev.psych.54.101601.145056 12172000

[B31] Fernández-Molina M. Bravo Castillo A. Fernández-Berrocal P. (2019). Profiles of perceived emotional intelligence in future preschool teachers: Implications for teacher education. *Rev. Electronica Interuniversitaria de Formacion Del Profesorado* 22 209–223. 10.6018/reifop.22.1.344131

[B32] Ferragut M. Fierro A. (2012). Emotional intelligence, well-being and academic achievement in preadolescents. *Revista Latinoam. Psicol.* 44 95–104. 10.14349/rlp.v44i3.1154

[B33] Flores M. A. (2006). Being a novice teacher in two different settings: Struggles, continuities, and discontinuities. *Teach. Coll. Rec.* 108 2021–2052. 10.1111/j.1467-9620.2006.00773.x

[B34] Hanley A. W. Palejwala M. H. Hanley R. T. Canto A. I. Garland E. L. (2015a). A failure in mind: Dispositional mindfulness and positive reappraisal as predictors of academic self-efficacy following failure. *Pers. Individ. Differ.* 86 332–337. 10.1016/j.paid.2015.06.033

[B35] Hanley A. Warner A. Garland E. L. (2015b). Associations between mindfulness, psychological well-being, and subjective well-being with respect to contemplative practice. *J. Happiness Stud.* 16 1423–1436. 10.1007/s10902-014-9569-5

[B36] Hassan A. Pheng K. F. Yew S. K. (2013). Philosophical perspectives on emotional intelligence, self efficacy and job satisfaction among secondary school teachers. *Int. J. Hum. Soc. Sci.* 3 109–114.

[B37] Hong J. Y. (2010). Pre-service and beginning teachers’ professional identity and its relation to dropping out of the profession. *Teach. Teach. Educ.* 26 1530–1543. 10.1016/j.tate.2010.06.003

[B38] Judge T. A. Jackson C. L. Shaw J. C. Scott B. A. Rich B. L. (2007). Self-efficacy and work-related performance: The integral role of individual differences. *J. Appl. Psychol.* 92 107–127. 10.1037/0021-9010.92.1.107 17227155

[B39] Kabat-Zinn J. (1990). *Full catastrophe living: Using the wisdom of your mind and body to face stress, pain, and illness.* New York, NY: Delacorte.

[B40] Kelly A. L. Berthelsen D. C. (1995). Preschool teachers’ experiences of stress. *Teach. Teach. Educ.* 11 345–357. 10.1016/0742-051X(94)00038-8

[B41] Kırkıç K. A. Çetinkaya F. (2020). The relationship between preschool teachers’ self-efficacy beliefs and their teaching attitudes. *Int. J. Eval. Res. Educ.* 9 807–815. 10.11591/ijere.v9i4.20670

[B42] Konstantopoulos S. (2014). Teacher effects, value-added models, and accountability. *Teach. Coll. Rec.* 116 10.1177/016146811411600109

[B43] Korthagen F. A. J. Evelein F. G. (2016). Relations between student teachers’ basic needs fulfillment and their teaching behavior. *Teach. Teach. Educ.* 60 234–244. 10.1016/j.tate.2016.08.021

[B44] Kostiæ-Bobanoviæ M. (2020). Perceived emotional intelligence and self-efficacy among novice and experienced foreign language teachers. *Econ. Res. Ekon. Istrazivanja* 33 1200–1213. 10.1080/1331677X.2019.1710232

[B45] Koydemir S. Şimşek ÖF. Schütz A. Tipandjan A. (2013). Differences in how trait emotional intelligence predicts life satisfaction: The role of affect balance versus social support in India and Germany. *J. Happiness Stud.* 14 51–66. 10.1007/s10902-011-9315-1

[B46] Lauermann F. König J. (2016). Teachers’ professional competence and wellbeing: Understanding the links between general pedagogical knowledge, self-efficacy and burnout. *Learn. Instr.* 45 9–19. 10.1016/j.learninstruc.2016.06.006

[B47] Law K. S. Wong C. S. Song L. J. (2004). The construct and criterion validity of emotional intelligence and its potential utility for management studies. *J. Appl. Psychol.* 89 483–496. 10.1037/0021-9010.89.3.483 15161407

[B48] Li Y. Zhang H. (2021). A study on the relations among work pressure, emotional intelligence, and subjective well-being of kindergarten teachers. *Rev. Cercetare Si Interventie Soc.* 73 22–33. 10.33788/rcis.73.2

[B49] Liu Y. Wang Z. Lü W. (2013). Resilience and affect balance as mediators between trait emotional intelligence and life satisfaction. *Pers. Individ. Differ.* 54 850–855. 10.1016/j.paid.2012.12.010

[B50] Loton D. J. Waters L. E. (2017). The mediating effect of self-efficacy in the connections between strength-based parenting, happiness and psychological distress in teens. *Front. Psychol.* 8:1707. 10.3389/fpsyg.2017.01707 29066986 PMC5641398

[B51] Luberto C. M. Cotton S. McLeish A. C. Mingione C. J. O’Bryan E. M. (2014). Mindfulness skills and emotion regulation: The mediating role of coping self-efficacy. *Mindfulness* 5 373–380. 10.1007/s12671-012-0190-6

[B52] Lyubomirsky S. King L. Diener E. (2005). The benefits of frequent positive affect: Does happiness lead to success? *Psychol. Bull.* 131 803–855. 10.1037/0033-2909.131.6.803 16351326

[B53] Ma Y. F. Sun J. M. (2009). “Subjective well-being in China: Dimension re-test, structure validity, and generalizability,” in *2009 International conference on management science and engineering–16th annual conference proceedings*, (Moscow: ICMSE), 10.1109/ICMSE.2009.5318149

[B54] Matiz A. Fabbro F. Paschetto A. Cantone D. Paolone A. R. Crescentini C. (2020). Positive impact of mindfulness meditation on mental health of female teachers during the COVID-19 outbreak in Italy. *Int. J. Environ. Res. Public Health* 17:6450. 10.3390/ijerph17186450 32899739 PMC7559290

[B55] Maxwell S. E. Cole D. A. (2007). Bias in cross-sectional analyses of longitudinal mediation. *Psychol. Methods* 12 23–44. 10.1037/1082-989X.12.1.23 17402810

[B56] Mayer J. D. Salovey P. (1997). “What is emotional intelligence?,” in *Emotional development and emotional intelligence implications for educators*, eds Salovey P. Sluyter D. (New York, NY: Basic Books).

[B57] Mayer D. Roberts R. D. Barsade S. G. (2008). Human abilities: Emotional intelligence. *Ann. Rev. Psychol.* 59 507–536. 10.1146/annurev.psych.59.103006.093646 17937602

[B58] McGrath B. J. Huntington A. D. (2007). The health and wellbeing of adults working in early childhood education. *Australas. J. Early Child.* 32 33–38. 10.1177/183693910703200306

[B59] Mérida-López S. Sánchez-Gómez M. Extremera N. (2020). Leaving the teaching profession: Examining the role of social support, engagement and emotional intelligence in teachers’ intentions to quit. *Psychosoc. Interv.* 29 141–151. 10.5093/PI2020A10

[B60] Mischel W. Shoda Y. (1995). A cognitive-affective system theory of personality: Reconceptualizing situations, dispositions, dynamics, and invariance in personality structure. *Psychol. Rev.* 102 246–268. 10.1037/0033-295X.102.2.246 7740090

[B61] Muijs D. Reynolds D. (2015). Teachers’ beliefs and behaviors: What really matters? *J. Classroom Interact.* 50 25–40.

[B62] Ngui G. K. Lay Y. F. (2019). The predicting roles of self-efficacy and emotional intelligence and the mediating role of resilience on subjective well-being: A PLS-SEM approach. *Pertanika J. Soc. Sci. Hum.* 27 1–25.

[B63] Pang D. Ruch W. (2019). Scrutinizing the components of mindfulness: Insights from current, past, and non-meditators. *Mindfulness* 10 492–505. 10.1007/s12671-018-0990-4

[B64] Phillips J. M. Gully S. M. (1997). Role of goal orientation, ability, need for achievement, and locus of control in the self-efficacy and goal-setting process. *J. Appl. Psychol.* 82 792–802. 10.1037/0021-9010.82.5.792

[B65] Podsakoff P. M. MacKenzie S. B. Lee J. Y. Podsakoff N. P. (2003). Common method biases in behavioral research: A critical review of the literature and recommended remedies. *J. Appl. Psychol.* 88 879–903. 10.1037/0021-9010.88.5.879 14516251

[B66] Rey L. Extremera N. Pena M. (2016). Emotional competence relating to perceived stress and burnout in Spanish teachers: A mediator model. *Peer J.* 4:e2087. 10.7717/peerj.2087 27280077 PMC4893324

[B67] Roberts B. W. Jackson J. J. (2008). Sociogenomic personality psychology. *J. Pers.* 76 1523–1544. 10.1111/j.1467-6494.2008.00530.x 19012657 PMC2699676

[B68] Rohrer J. M. Lucas R. E. (2020). Causal effects of well-being on health: It’ s complicated. *PsyArXiv* 2017 1–24.

[B69] Sahdra B. Ciarrochi J. Parker P. (2016). Nonattachment and mindfulness: Related but distinct constructs. *Psychol. Assess.* 28 819–829. 10.1037/pas0000264 27078180

[B70] Schaufeli W. B. Taris T. W. (2014). “A critical review of the job demands-resources model: Implications for improving work and health,” in *Bridging occupational, organizational and public health: A transdisciplinary approach*, Vol. 9789400756 10.1007/978-94-007-5640-3_4

[B71] Scholz U. Doña B. G. Sud S. Schwarzer R. (2002). Is general self-efficacy a universal construct? Psychometric findings from 25 countries. *Eur. J. Psychol. Assess.* 18 242–251. 10.1027//1015-5759.18.3.242

[B72] Schutte N. S. Malouff J. M. (2011). Emotional intelligence mediates the relationship between mindfulness and subjective well-being. *Pers. Individ. Differ.* 50 1116–1119. 10.1016/j.paid.2011.01.037

[B73] Shier M. L. Graham J. R. (2011). Mindfulness, subjective well-being, and social work: Insight into their interconnection from social work practitioners. *Soc. Work Educ.* 30 29–44. 10.1080/02615471003763188

[B74] Sinclair H. Feigenbaum J. (2012). Trait emotional intelligence and borderline personality disorder. *Pers. Individ. Differ.* 52 674–679. 10.1016/j.paid.2011.12.022

[B75] Skaalvik E. M. Skaalvik S. (2011). Teacher job satisfaction and motivation to leave the teaching profession: Relations with school context, feeling of belonging, and emotional exhaustion. *Teach. Teach. Educ.* 27 1029–1038.

[B76] Spencer T. D. Detrich R. Slocum T. A. (2012). Evidence-based practice: A framework for making effective decisions. *Educ. Treat. Child.* 35 127–151. 10.1353/etc.2012.0013 34409987

[B77] Sumsion J. (2002). Becoming, being and unbecoming an early childhood educator: A phenomenological case study of teacher attrition. *Teach. Teach. Educ.* 18 869–885. 10.1016/S0742-051X(02)00048-3

[B78] Thoms P. Moore K. S. Scott K. S. (1996). The relationship between self-efficacy for participating in self-managed work groups and the big five personality dimensions. *J. Organ. Behav.* 17 349–362. 10.1002/(SICI)1099-1379(199607)17:4<349::AID-JOB756<3.0.CO;2-3

[B79] Tschannen-Moran M. Hoy A. W. (2007). The differential antecedents of self-efficacy beliefs of novice and experienced teachers. *Teach. Teach. Educ.* 23 944–956. 10.1016/j.tate.2006.05.003

[B80] Tsui A. S. Ashford S. J. Clair L. S. T. Xin K. R. (1995). Dealing with discrepant expectations: Response strategies and managerial effectiveness. *Acad. Manage. J.* 38 1515–1543. 10.5465/256842 256842

[B81] Valente S. Lourenço A. A. (2020). Conflict in the classroom: How teachers’ emotional intelligence influences conflict management. *Front. Educ.* 5:5. 10.3389/feduc.2020.00005

[B82] Walker C. O. Greene B. A. Mansell R. A. (2006). Identification with academics, intrinsic/extrinsic motivation, and self-efficacy as predictors of cognitive engagement. *Learn. Individ. Differ.* 16 1–12.

[B83] Wells M. B. (2015). Predicting preschool teacher retention and turnover in newly hired head start teachers across the first half of the school year. *Early Child. Res. Q.* 30 152–159. 10.1016/j.ecresq.2014.10.003

[B84] Wong C. S. Law K. S. (2002). The effects of leader and follower emotional intelligence on performance and attitude: An exploratory study. *Leadersh. Q.* 13 243–274. 10.1016/S1048-9843(02)00099-1

[B85] Wu Y. Lian K. Hong P. Liu S. Lin R. M. Lian R. (2019). Teachers’ emotional intelligence and self-efficacy: Mediating role of teaching performance. *Soc. Behav. Pers.* 47 1–10. 10.2224/sbp.7869

[B86] Yurayat P. Seechaliao T. (2021). Effectiveness of online positive psychology intervention on psychological well-being among undergraduate students. *J. Educ. Learn.* 10 143–155. 10.5539/jel.v10n4p143

[B87] Zhang J. Schwarzer R. (1995). Measuring optimistic self-beliefs–a chinese adaptation of the general self-efficacy scale. *Psychol. Int. J. Psychol. Orient* 38 174–181.

